# Virologic, Lipid, Renal and Hepatic Laboratory Outcomes After Switching to Doravirine/Lamivudine/Tenofovir Disoproxil Fumarate in Treatment-Experienced People with HIV: Real-World Evidence from the SHINe-SHIC Cohort

**DOI:** 10.3390/idr18050105

**Published:** 2026-09-17

**Authors:** Manuela Ceccarelli, Emmanuele Venanzi Rullo, Andrea Marino, Andrea De Vito, Cristina Micali, Serena Spampinato, Ylenia Russotto, Antonio Albanese, Maria Chiara Frasca, Sonia Agata Sofia, Giovanni Francesco Pellicanò, Benedetto Maurizio Celesia, Paolo Maggi, Giordano Madeddu, Giuseppe Nunnari

**Affiliations:** 1 Unit of Infectious Diseases, Department of Medicine and Surgery, “Kore” University of Enna, 94100 Enna, Italy; paolo.maggi@unikore.it; 2 Unit of Infectious Diseases, Department of Clinical and Experimental Medicine, University of Messina, 98125 Messina, Italy; emmanuele.venanzirullo@unime.it (E.V.R.); ylenia.russotto@unime.it (Y.R.); giovanni.pellicano@unime.it (G.F.P.); 3 Unit of Infectious Diseases, Department of Clinical and Experimental Medicine, University of Catania, 95123 Catania, Italy; andrea.marino@unict.it (A.M.); serenaspampinato93@gmail.com (S.S.); bmcelesia@gmail.com (B.M.C.); giuseppe.nunnari1@unict.it (G.N.); 4 Unit of Infectious Diseases, Azienda Ospedaliera Universitaria di Sassari, Department of Medicine, Surgery and Pharmacy, University of Sassari, 07100 Sassari, Italy; andreadevitoaho@gmail.com (A.D.V.); giordano@uniss.it (G.M.); 5 Emory National Primate Research Center, Department of Pathology and Laboratory Medicine, Emory University, Atlanta, GA 30329, USA; 6 Unit of Infectious Diseases, Azienda Ospedaliera “Papardo”, 98158 Messina, Italy; antonioalbanese@icloud.com; 7 Unit of Infectious Diseases, Azienda Ospedaliera Universitaria Policlinico “G. Rodolico—San Marco”, 95121 Catania, Italy; m.chiarafrasca@yahoo.it; 8 Unit of Infectious Diseases, Azienda Ospedaliera per l’Emergenza “Cannizzaro”, 95126 Catania, Italy; sonia.sofia@email.it

**Keywords:** HIV, doravirine, lamivudine, tenofovir disoproxil fumarate, DOR/3TC/TDF, treatment switch, lipid profile, renal function, real-world evidence

## Abstract

**Background/Objectives**: Doravirine/lamivudine/tenofovir disoproxil fumarate (DOR/3TC/TDF) is used as a switch regimen in treatment-experienced people with HIV (PWH), particularly when simplification, lipid improvement, or avoidance of interaction-prone regimens is needed. We evaluated 48-week virologic effectiveness and laboratory changes after switching to DOR/3TC/TDF in routine care. **Methods**: This multicenter retrospective study included adults with HIV who switched to fixed-dose DOR/3TC/TDF at seven Italian HIV centers within the Sardinian HIV Network-Sicilian HIV Cohort (SHINe-SHIC) network. The switch visit served as baseline; follow-up data were extracted at 24 and 48 weeks. The primary endpoint was HIV RNA < 50 copies/mL at 48 weeks in an observed analysis. Secondary endpoints were changes in lipid and lipid-derived parameters, renal function, and hepatic laboratory markers. **Results**: Ninety-eight participants were included; 75 (76.5%) were male, and the median age was 52.4 years. At 48 weeks, 72/81 participants (88.9%) had HIV RNA < 50 copies/mL and 78/81 (96.3%) had HIV RNA < 200 copies/mL. Total cholesterol decreased from 199 to 165 mg/dL (median paired change, −22 mg/dL; *p* < 0.001), low-density lipoprotein cholesterol from 118 to 106.5 mg/dL (−13.5 mg/dL; *p* = 0.001), and triglycerides from 112.5 to 94 mg/dL (−10.5 mg/dL; *p* = 0.021). Non-high-density lipoprotein cholesterol and the total cholesterol/high-density lipoprotein cholesterol ratio improved, whereas high-density lipoprotein cholesterol decreased modestly. Serum creatinine and estimated glomerular filtration rate remained stable. Alanine aminotransferase increased modestly; aspartate aminotransferase and gamma-glutamyl transferase did not significantly change. **Conclusions**: Switching to DOR/3TC/TDF maintained virologic control, improved lipid and lipid-derived parameters, and was not associated with renal function decline among participants with follow-up data. In selected treatment-experienced PWH, DOR/3TC/TDF may be useful when lipid improvement, simplification, or management of drug-drug interaction concerns are treatment goals.

## 1. Introduction

As antiretroviral treatment (ART) has transformed HIV infection into a chronic condition, treatment decisions in people with HIV (PWH) are no longer limited to maintaining virologic suppression. In routine care, regimen optimization also has to account for cumulative toxicity, drug-drug interactions, cardiovascular and metabolic risk, renal safety, and the broader comorbidity burden of each patient [1,2,3,4]. Switch strategies that preserve antiviral effectiveness while improving long-term tolerability, lipid profile, and laboratory safety therefore remain an important part of HIV care [5].

Lipid abnormalities remain a major concern in the long-term care of PWH, particularly in aging treatment-experienced populations in whom chronic inflammation, traditional cardiovascular risk factors, comorbidities, and cumulative ART exposure often overlap [3,4,5,6]. Metabolic changes reported after ART initiation or switch, particularly with contemporary ART regimens, have increased attention to the metabolic profile of optimization strategies [7,8,9]. In routine clinical practice, lipid parameters and lipid-derived markers are among the most consistently available measures to evaluate the early metabolic impact of ART switch strategies.

Doravirine (DOR), a next-generation non-nucleoside reverse transcriptase inhibitor (NNRTI), is a useful anchor for switch strategies because it avoids booster-related interactions, food requirements, and gastric acid–related restrictions, while retaining activity against several common NNRTI-associated substitutions [10,11,12]. Clinical trial data support the virologic durability and tolerability of DOR-based regimens, including doravirine/lamivudine/tenofovir disoproxil fumarate (DOR/3TC/TDF) as a switch strategy in adults with virologic suppression [13,14]. Beyond virologic efficacy, DOR-based therapy has shown favorable lipid changes compared with boosted protease inhibitor- or efavirenz-based comparators [9,15]. The fixed-dose DOR/3TC/TDF regimen is therefore clinically interesting because it combines an unboosted DOR anchor with a tenofovir disoproxil fumarate (TDF) backbone, which may add lipid benefit but still requires careful renal and bone safety assessment [16,17].

In this multicenter retrospective real-world cohort, we studied treatment-experienced PWH who switched to fixed-dose DOR/3TC/TDF in routine clinical practice. We focused first on virologic effectiveness at 48 weeks. We then assessed changes in lipid and lipid-derived parameters, renal function, and hepatic laboratory markers after switch, while also describing immunologic parameters. Because prior ART may influence both lipid and renal trajectories, we also explored changes according to the previous ART class and previous nucleoside/nucleotide reverse transcriptase inhibitor (NRTI) backbone.

## 2. Materials and Methods

### 2.1. Study Design and Population

This multicenter retrospective observational study included treatment-experienced adults with HIV who switched to fixed-dose DOR/3TC/TDF in routine clinical practice between June 2020 and May 2022. Data were collected from HIV centers within the SHINe-SHIC network. The date of switch to DOR/3TC/TDF was used as the index date. Follow-up data were extracted from routine clinical visits performed approximately 24 and 48 weeks after switch, corresponding to the 6-month and 12-month follow-up assessments.

### 2.2. Eligibility Criteria

Eligible participants were adults aged ≥18 years with documented HIV infection, previous ART exposure, and initiation of fixed-dose DOR/3TC/TDF in routine care. Inclusion required available demographic, clinical, and laboratory data at the switch visit and at least one follow-up assessment. Participants were excluded when key information on treatment regimen or follow-up status was unavailable.

### 2.3. Data Collection and Definitions

Collected variables included age, sex, participating center, years since HIV diagnosis, comorbidities, hepatitis B virus (HBV) and hepatitis C virus (HCV) status, smoking status, previous ART regimen, ART class before switch, reason for switching, and concomitant antiretroviral drugs used with DOR/3TC/TDF. Documented hepatitis B surface antigen (HBsAg) positivity was used to define HBV status. The HCV status variable reflected current or previous HCV infection and/or positive HCV serology according to available clinical records and should not be interpreted as necessarily active HCV infection.

Laboratory data were recorded at the switch visit and at approximately 24 and 48 weeks, including HIV RNA, CD4+ and CD8+ T-cell counts and percentages, CD4/CD8 ratio, serum creatinine, estimated glomerular filtration rate (eGFR), aspartate aminotransferase (AST), alanine aminotransferase (ALT), gamma-glutamyl transferase (GGT), total cholesterol, high-density lipoprotein cholesterol (HDL-C), low-density lipoprotein cholesterol (LDL-C), and triglycerides. The eGFR was calculated using the creatinine-based CKD-EPI 2021 equation; cystatin C was not available in the dataset. Non-HDL cholesterol was calculated as total cholesterol minus HDL-C. Lipid-derived ratios included total cholesterol/HDL-C, LDL-C/HDL-C, and triglycerides/HDL-C. The switch visit was used as baseline for all longitudinal analyses.

### 2.4. Study Endpoints

The primary endpoint was virologic effectiveness at 48 weeks, assessed as the proportion of participants with available HIV RNA < 50 copies/mL in an observed analysis. The threshold of HIV RNA < 200 copies/mL was used as a supportive virologic threshold. Secondary endpoints were changes from the switch visit to 24 and 48 weeks in lipid and lipid-derived parameters, renal function, hepatic laboratory markers, and immunologic parameters. Renal assessment included serum creatinine and eGFR; hepatic laboratory assessment included AST, ALT, and GGT. Lipid assessment included total cholesterol, LDL-C, HDL-C, triglycerides, non-HDL cholesterol, and lipid ratios. Exploratory analyses described renal and lipid changes according to previous ART class, categorized as integrase strand transfer inhibitor (INSTI)-, non-nucleoside reverse transcriptase inhibitor (NNRTI)-, protease inhibitor (PI)-based therapy, or unknown. Additional exploratory analyses described lipid changes according to previous nucleoside/nucleotide reverse transcriptase inhibitor (NRTI) backbone, categorized as tenofovir alafenamide (TAF)-containing, abacavir/lamivudine (ABC/3TC)-containing, tenofovir disoproxil fumarate (TDF)-containing, or unknown/not classifiable.

### 2.5. Statistical Analysis

Continuous variables were summarized as medians with interquartile ranges (IQRs), and categorical variables as counts and percentages. Because several laboratory variables were non-normally distributed and included outlying values, within-participant changes were analyzed using the Wilcoxon signed-rank test. The main paired comparison was between the switch visit and 48 weeks; additional comparisons assessed changes from switch to 24 weeks and from 24 to 48 weeks. Missing data were not imputed. Virologic outcomes were analyzed using an observed approach, including participants with available HIV RNA measurements at each time point. For paired analyses, only participants with available measurements at both compared time points were included; therefore, the number of paired observations varied across variables. Data availability was summarized at the switch visit, 24 weeks, and 48 weeks for the main virologic and laboratory variables.

Exploratory analyses by previous ART class and previous NRTI backbone were based on median paired changes from the switch visit to 48 weeks, with IQRs. These analyses were interpreted descriptively because of the limited number of paired observations within strata. Graphical analyses by previous ART class were restricted to INSTI-, NNRTI-, and PI-based regimens. The supplementary graphical analysis by previous NRTI backbone focused on the two main informative switch groups, TAF-containing and ABC/3TC-containing regimens.

No adjustment for multiple comparisons was applied because secondary and subgroup analyses were exploratory and involved clinically related laboratory domains rather than independent confirmatory hypotheses. Therefore, *p*-values for secondary outcomes were interpreted descriptively, together with the magnitude and direction of the observed changes.

All statistical tests were two-sided, and *p*-values < 0.05 were considered statistically significant. Analyses were performed using R version 4.5.3 (R Foundation for Statistical Computing, Vienna, Austria) with the open-source readxl, dplyr, tidyr, purrr, readr, broom, and ggplot2 packages.

### 2.6. Ethical Approval

The study was conducted in accordance with the Declaration of Helsinki. The SHINe-SHIC cohort was approved by the Ethics Committee of the University of Messina (protocol code 34/17, 22 March 2017; approval date 22 May 2017). Each participating center submitted the cohort protocol to its local ethics committee according to institutional requirements. Written informed consent was obtained from all participants included in the study.

### 2.7. AI Disclosure

A large language model (ChatGPT, GPT-5.5; OpenAI, San Francisco, CA, USA) was used during manuscript preparation to assist with language editing, clarity, and organization of the text. It was not used to generate original data. All analyses, results, and manuscript content were critically reviewed and validated by the authors, who take full responsibility for the final work.

## 3. Results

### 3.1. Study Population and Baseline Characteristics

Ninety-eight treatment-experienced PWH who switched to fixed-dose DOR/3TC/TDF between June 2020 and May 2022 were included in the analysis. Baseline was defined as the switch date. Overall, 75 participants were male (76.5%), and median age was 52.4 years (IQR, 43.0–58.8). Median time since HIV diagnosis was 14 years (IQR, 9–23). At switch, median CD4+ T-cell count was 730 cells/µL (IQR, 503.5–928.5), and median eGFR was 95 mL/min/1.73 m^2^ (IQR, 81–101). The most common comorbidities were hypertension (28.6%), hypercholesterolemia (16.3%), diabetes mellitus (12.2%), obesity (7.1%), and chronic kidney disease (4.1%). Documented HBsAg positivity was present in 17.3% of participants. Current or previous HCV infection and/or positive HCV serology was reported in 7.1% of participants and should not be interpreted as necessarily active HCV infection. Baseline characteristics are shown in Table 1.

Data availability at the switch visit, 24 weeks, and 48 weeks is reported in Table S1. HIV RNA measurements were available for 95 participants at the switch visit, 74 at 24 weeks, and 81 at 48 weeks. The number of paired observations available for longitudinal laboratory analyses varied by parameter, reflecting the retrospective routine-care design.

### 3.2. Previous ART Regimens and Reasons for Switch

Before switching to DOR/3TC/TDF, 41 participants (41.8%) were on an INSTI-based regimen, 27 (27.6%) on an NNRTI-based regimen, and 27 (27.6%) on a PI-based regimen. All participants were treatment-experienced; however, the immediately preceding ART class could not be classified from available records in 3 participants (3.1%). The most frequent previous regimens were DTG/ABC/3TC and DRV/c/FTC/TAF, each used by 26 participants (26.5%), followed by NVP + ABC/3TC in 19 participants (19.4%). Previous NRTI backbone was TAF-containing in 46 participants (46.9%), ABC/3TC-containing in 46 participants (46.9%), TDF-containing in 3 participants (3.1%), and unknown or not classifiable in 3 participants (3.1%).

The main reasons for switching were ART simplification (28/98, 28.6%), pre-emptive switch (27/98, 27.6%), and drug–drug interactions (22/98, 22.4%). Toxicity or adverse reactions to previous ART accounted for 9 switches (9.2%), dyslipidemia for 5 (5.1%), and other reasons for 6 (6.1%); the reason for switch was missing in 1 participant. Previous ART regimens and reasons for switching are reported in Table 2 and Table 3.

### 3.3. Virologic and Immunologic Outcomes

At the switch visit, 83/95 participants (87.4%) had HIV RNA < 50 copies/mL and 92/95 (96.8%) had HIV RNA < 200 copies/mL. In the observed analysis, 65/74 participants (87.8%) had HIV RNA < 50 copies/mL at 24 weeks and 72/81 (88.9%) at 48 weeks. Using the supportive threshold of HIV RNA < 200 copies/mL, suppression was documented in 73/74 participants (98.6%) at 24 weeks and 78/81 (96.3%) at 48 weeks. Three participants had HIV RNA ≥ 200 copies/mL at the 48-week assessment. All three had HIV RNA < 200 copies/mL at the switch visit; two had switched from PI-based regimens including DRV/c/FTC/TAF, and one from an INSTI-based DTG/ABC/3TC regimen. All three were again virologically suppressed at the subsequent follow-up without ART modification, supporting intermittent or suboptimal adherence rather than confirmed treatment failure. Immunologic parameters remained stable from switch to 48 weeks, with no significant changes in CD4+ or CD8+ T-cell counts and percentages or in CD4/CD8 ratio (Table 4).

### 3.4. Renal and Hepatic Laboratory Safety

Renal parameters did not show significant changes from switch to 48 weeks. Serum creatinine was 0.9 mg/dL (IQR 0.8–1.0) at switch and 0.9 mg/dL (IQR 0.8–1.1) at 48 weeks, with a median paired change of 0 mg/dL (IQR −0.095 to 0.035; *p* = 0.221). The eGFR was also unchanged (94 mL/min/1.73 m^2^ [IQR 74–98] vs. 93.2 mL/min/1.73 m^2^ [IQR 77.8–100]; median paired change, 0 mL/min/1.73 m^2^ [IQR −2 to 7.5]; *p* = 0.099).

Among hepatic laboratory parameters, ALT increased modestly but significantly, from 25 IU/L (IQR 22–38) at switch to 31 IU/L (IQR 22–55) at 48 weeks, with a median paired change of 6 IU/L (IQR −4 to 14; *p* = 0.008). Using an ALT upper limit of normal of 40 IU/L, grade ≥ 2 ALT elevation, defined as ≥2.5 times the upper limit of normal, was present in 2/71 participants (2.8%) at the switch visit and 5/54 participants (9.3%) at 48 weeks. The AST and GGT did not significantly change.

### 3.5. Lipid and Lipid-Derived Outcomes

Lipid parameters improved after switching to DOR/3TC/TDF. At 48 weeks, total cholesterol decreased from 199 to 165 mg/dL (median paired change, −22 mg/dL; IQR, −52 to 1; *p* < 0.001), LDL-C from 118 to 106.5 mg/dL (median paired change, −13.5 mg/dL; IQR −29.75 to 5.8; *p* = 0.001), and triglycerides from 112.5 to 94 mg/dL (median paired change, −10.5 mg/dL; IQR −54 to 13.75; *p* = 0.021).

High-density lipoprotein cholesterol also decreased modestly, from 48 to 44 mg/dL (median paired change, −3 mg/dL; IQR −9 to 2; *p* = 0.022). Non-HDL cholesterol and the total cholesterol/HDL-C and triglycerides/HDL-C ratios improved significantly, whereas the LDL-C/HDL-C ratio did not significantly change. Overall, these changes suggest an overall favorable shift in the lipid profile despite the modest decrease in HDL-C.

### 3.6. Changes at 24 Weeks

Lipid improvements were already evident at 24 weeks (Table S2). From 24 to 48 weeks, lipid and lipid-derived parameters showed no further significant changes (Table S3), indicating that most of the lipid benefit occurred during the first 24 weeks and was then maintained through 48 weeks.

### 3.7. Exploratory Analyses by Previous ART Class and NRTI Backbone

**Figure 1:**
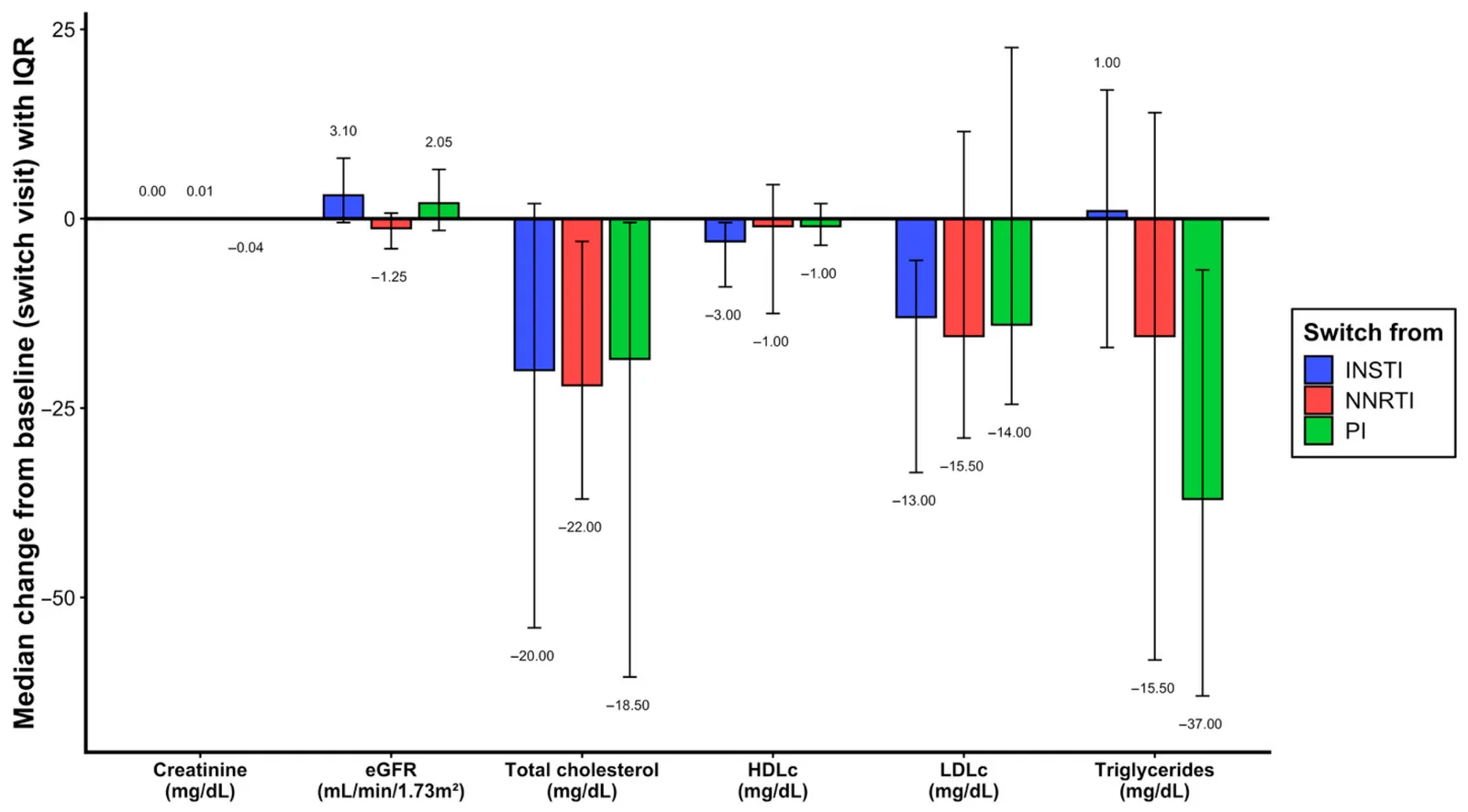
Median paired changes from switch visit to 48 weeks in eGFR and lipid parameters according to previous ART class. Bars represent median changes and error bars represent interquartile ranges (IQR). Values above or below bars indicate median change. The previous ART classes were categorized as INSTI-, NNRTI-, or PI-based regimens. Creatinine changes are reported in Table 4. Abbreviations: eGFR, estimated glomerular filtration rate; HDLc, high-density lipoprotein cholesterol; INSTI, integrase strand transfer inhibitor; LDLc, low-density lipoprotein cholesterol; NNRTI, non-nucleoside reverse transcriptase inhibitor; PI, protease inhibitor.

In exploratory analyses stratified by previous ART class, renal parameters showed no clinically relevant deterioration across INSTI-, NNRTI-, or PI-based switch groups, with median eGFR changes ranging from −1.25 to +3.1 mL/min/1.73 m^2^. Lipid reductions were observed across previous ART classes. Total cholesterol and LDL-C decreased in all three groups, whereas triglyceride reductions appeared more pronounced among participants switching from PI-based regimens. Given the limited paired sample size within strata, no formal comparative estimates were calculated. Median paired changes by previous ART class are shown in Figure 1.

A complementary descriptive analysis according to the previous NRTI backbone is reported in Table S4 and Figure S1. The graphical analysis focused on the two main informative switch groups, TAF-containing and ABC/3TC-containing backbones, both switching to TDF-containing DOR/3TC/TDF. Lipid reductions were observed in both groups, with numerically larger reductions in several lipid and lipid-derived parameters among participants switching from TAF-containing regimens. Participants previously receiving TDF-containing regimens were retained only in the Supplementary Table S4 for completeness, because no TDF exposure change occurred in this subgroup and the sample size was very small.

## 4. Discussion

In this multicenter real-world cohort of treatment-experienced PWH switching to fixed-dose DOR/3TC/TDF, virologic control was maintained in most participants through 48 weeks, and renal function remained stable. After the switch, total cholesterol, LDL-C, triglycerides, non-HDL cholesterol, and lipid-derived ratios decreased significantly. The HDL-C level also decreased modestly, but this occurred alongside reductions in atherogenic lipid fractions and total cholesterol/HDL-C ratio. Overall, these findings support DOR/3TC/TDF as a switch option for selected treatment-experienced PWH, particularly when lipid improvement or avoidance of boosted or otherwise metabolically unfavorable regimens is part of the treatment rationale [13,14,18,19,20,21].

Our results fit within a growing real-world experience with DOR-based regimens in treatment-experienced PWH. Italian cohorts, including SCOLTA analyses, have described DOR-based switches driven by simplification, dyslipidemia, toxicity prevention, drug-drug interaction concerns, comorbidities, or polypharmacy, and have reported lipid and hepatic laboratory outcomes after switch [22,23,24,25,26,27,28]. Beyond the Italian setting, recent real-world studies from China, Serbia, and broader European clinical practice have also reported early effectiveness and favorable lipid or laboratory safety outcomes with DOR/3TC/TDF in cohorts including treatment-experienced PWH [21,29,30]. Our cohort adds to this literature by focusing on fixed-dose DOR/3TC/TDF and by combining paired lipid-derived, renal, and hepatic laboratory assessments with exploratory stratification by previous ART class and NRTI backbone.

Virologic outcomes were consistent with the use of DOR/3TC/TDF as a switch regimen in treatment-experienced PWH [13,14]. Most participants maintained HIV RNA < 50 copies/mL through 48 weeks, and suppression below the supportive threshold of 200 copies/mL was documented in 96.3% of those with available HIV RNA at 48 weeks. The few episodes of HIV RNA ≥ 200 copies/mL did not lead to ART modification, and subsequent resuppression supported intermittent or suboptimal adherence rather than confirmed treatment failure. These findings should nevertheless be interpreted within an observed analysis, because missing HIV RNA measurements and the retrospective design limit formal estimation of virologic failure risk [20,21,31].

The main metabolic finding in our cohort was the improvement in lipid and lipid-derived parameters after switch. Total cholesterol, LDL-C, triglycerides, and non-HDL cholesterol decreased significantly at 48 weeks. The HDL-C level also decreased modestly, but this occurred alongside reductions in atherogenic lipid fractions and ratios, suggesting an overall favorable lipid shift. Lipid improvements were already present at 24 weeks and did not further increase between 24 and 48 weeks, consistent with an early effect that was maintained through the first year. Because switches were clinically driven, these changes should be interpreted as outcomes of a targeted real-world switch strategy rather than as evidence of an isolated doravirine-specific lipid effect.

These findings are in line with pivotal DOR studies showing favorable lipid changes compared with boosted protease inhibitor- or efavirenz-based comparators, and with Italian real-world DOR-switch cohorts and SCOLTA analyses reporting lipid and hepatic outcomes after DOR-based switches [15,22,23,24,25,26,27,28,32,33]. They are also clinically plausible because both the unboosted DOR anchor and the TDF backbone may contribute to lipid improvement, with TDF consistently associated with a more favorable lipid profile than TAF in switch studies [16,17,18,34,35].

Renal monitoring remains important when using TDF-containing regimens, especially in aging treatment-experienced PWH. In our cohort, serum creatinine and eGFR remained stable through 48 weeks, with no signal of renal function decline after switch to DOR/3TC/TDF. This is important because TDF may offer lipid advantages compared with TAF, but its use still requires careful patient selection and follow-up [17]. Our results are in line with real-world studies of DOR/3TC/TDF reporting stable creatinine or eGFR in selected patients [17,18,20,36].

The proportion of participants with documented HBsAg positivity also deserves clinical consideration. In this subgroup, the TDF/3TC backbone provides HBV activity and may have favored selection of a TDF-containing regimen, provided renal and bone risks were considered acceptable. However, HBV status was not systematically captured as a reason for switch, and this retrospective dataset does not allow us to determine whether documented HBsAg positivity influenced individual prescribing decisions.

Among hepatic laboratory markers, ALT increased modestly but significantly at 48 weeks, whereas AST and GGT did not change. This isolated ALT increase should be interpreted cautiously. Potential contributors include background liver disease, metabolic dysfunction-associated steatotic liver disease, viral hepatitis status, alcohol use, concomitant medications, and routine biological variability, none of which could be fully characterized in this retrospective dataset. Available trial and real-world data have not identified a consistent hepatotoxicity signal with DOR/3TC/TDF; therefore, the observed ALT change should be considered a laboratory finding requiring clinical contextualization rather than evidence of treatment-related hepatic toxicity. Therefore, the observed ALT change should be considered a laboratory finding requiring clinical contextualization rather than evidence of treatment-related hepatic toxicity. Previous real-world studies of DOR/3TC/TDF have generally reported stable liver enzymes or no major hepatic safety signals in treatment-experienced PWH, while studies with standardized adverse event reporting, such as DORA, are better suited to define the clinical relevance of these laboratory changes [20,21,37].

In the exploratory analysis by the previous ART class, renal parameters remained stable across INSTI-, NNRTI-, and PI-based switch groups. Lipid reductions were seen in all three groups, with triglyceride decreases appearing more marked among participants switching from PI-based regimens. This is plausible given the known lipid effects of boosted PI-based therapy and the favorable lipid profile observed with DOR compared to boosted darunavir in DRIVE-FORWARD [15,19,33]. Similar lipid improvements have also been described in real-world switch studies of DOR/3TC/TDF, including cohorts with many participants switching from INSTI-based regimens [18,20]. These subgroup results remain descriptive, given the small paired samples within strata.

Clinically, these data support DOR/3TC/TDF as a possible targeted switch regimen for treatment-experienced PWH in whom virologic control must be maintained while addressing lipid abnormalities, simplification needs, or drug–drug interaction concerns [1,2]. This is consistent with current guideline recommendations to individualize ART according to comorbidities and long-term treatment burden. The regimen may be particularly useful when switching from boosted PI-based regimens or other therapies associated with lipid or metabolic concerns [15,18,19,20,33,36]. Because of the TDF backbone, however, DOR/3TC/TDF should be considered in patients with an appropriate renal and bone safety profile rather than used as a universal switch approach [17].

Several limitations should be acknowledged. The retrospective observational design limits causal interpretation and introduces missing data, selection bias, and variability in follow-up across centers. The study was not designed to isolate the independent pharmacologic effect of doravirine from the broader effect of a clinically driven switch to an unboosted, TDF-containing regimen, and the absence of a control group prevents direct comparison with other contemporary switch strategies. Clinical adverse events, toxicity grading, treatment discontinuations, reasons for discontinuation, and detailed concomitant medication changes were not systematically captured; therefore, safety, treatment persistence, and real-world tolerability could not be fully assessed. Initiation, discontinuation, or dose changes of lipid-lowering therapy during follow-up were also not systematically collected, so unmeasured treatment changes may have contributed to lipid improvements. Bone health was not systematically assessed, including bone mineral density, fragility fracture history, vitamin D status, and incident bone events; therefore, this study cannot evaluate bone safety after switching to TDF-containing DOR/3TC/TDF. Race/ethnicity was homogeneous, with all participants reported as White, limiting generalizability to more diverse populations. Historical resistance data and genotypic resistance testing during episodes of viremia were not systematically available, limiting interpretation of the few cases with HIV RNA ≥ 200 copies/mL during follow-up. Finally, subgroup analyses by previous ART class and NRTI backbone were exploratory and limited by small paired samples; these results should be viewed as descriptive.

The study also has strengths. It reflects routine care across multiple HIV centers and includes treatment-experienced PWH with long-standing infection and a relevant comorbidity burden. By using paired lipid fractions, lipid-derived markers, renal function, and hepatic laboratory parameters, the study provides a practical assessment of DOR/3TC/TDF in a real-world switch setting.

## 5. Conclusions

In conclusion, switching to fixed-dose DOR/3TC/TDF in treatment-experienced PWH maintained virologic control through 48 weeks, improved lipid and lipid-derived parameters, and did not show evidence of renal function decline among participants with available follow-up. Alanine aminotransferase increased modestly, without parallel changes in AST or GGT. In appropriately selected patients, DOR/3TC/TDF may be a useful targeted switch option when lipid improvement, simplification, or management of drug-drug interaction concerns are treatment goals. These findings support its real-world positioning of DOR/3TC/TDF as a targeted switch regimen rather than a universal optimization strategy, although treatment persistence and discontinuation risk could not be assessed in this study.

## Figures and Tables

**Table 1:** Baseline characteristics of study participants.

Characteristics	Overall Cohort
*N*	98
Age, years; median (IQR)	52.4 (43–58.8)
Male sex; *n* (%)	75 (76.5%)
Race/ethnicity, white; *n* (%)	98 (100%)
Years since HIV diagnosis; median (IQR)	14 (9–23)
Hypertension; *n* (%)	28 (28.6%)
Hypercholesterolemia; *n* (%)	16 (16.3%)
Diabetes mellitus; *n* (%)	12 (12.2%)
Obesity; *n* (%)	7 (7.1%)
Chronic kidney disease *; *n* (%)	4 (4.1%)
Documented HBsAg positivity; *n* (%)	17 (17.3%)
Current or previous HCV infection/positive HCV serology; *n* (%)	7 (7.1%)
Smoking; *n* (%)	20 (20.4%)
HIV RNA at switch, copies/mL; median (IQR)	0 (0–21.5)
CD4+ T-cell count at switch, cells/µL; median (IQR)	730 (503.5–928.5)
eGFR at switch, mL/min/1.73 m^2^; median (IQR)	95 (81–101)
Total cholesterol at switch, mg/dL; median (IQR)	199 (162–233)
LDL-C at switch, mg/dL; median (IQR)	119 (88–156.5)
Triglycerides at switch, mg/dL; median (IQR)	116 (76–165)

* Chronic kidney disease was recorded as a clinical comorbidity based on medical history. Detailed CKD staging and proteinuria data were not systematically available. Abbreviations: CD4, cluster of differentiation 4; eGFR, estimated glomerular filtration rate; HBsAg, hepatitis B surface antigen; HCV, hepatitis C virus; HIV, human immunodeficiency virus; IQR, interquartile range; LDL-C, low-density lipoprotein cholesterol.

**Table 2:** Previous ART regimens.

Last Regimen Before the Switch	N	%
DTG/ABC/3TC	26	26.5
DRV/c/FTC/TAF	26	26.5
NVP + ABC/3TC	19	19.4
RAL + FTC/TAF	6	6.1
BIC/FTC/TAF	5	5.1
RPV/FTC/TAF	5	5.1
EVG/c/FTC/TAF	4	4.1
EFV/FTC/TDF	3	3.1
ATV + ABC/3TC	1	1
Missing	3	3.1

Abbreviations: ABC, abacavir; ART, antiretroviral therapy; ATV, atazanavir; BIC, bictegravir; DRV/c, darunavir/cobicistat; DTG, dolutegravir; EFV, efavirenz; EVG/c, elvitegravir/cobicistat; FTC, emtricitabine; NVP, nevirapine; RAL, raltegravir; RPV, rilpivirine; TAF, tenofovir alafenamide; TDF, tenofovir disoproxil fumarate; 3TC, lamivudine.

**Table 3:** Reasons for switching.

Reason	N	%
Toxicity	5	5.1
Simplification	28	28.6
ADR	4	4.1
Pre-emptive	27	27.6
DDI	22	22.4
Dyslipidemia	5	5.1
Other	6	6.1
Missing	1	1

Abbreviations: ADR, adverse drug reaction; DDI, drug–drug interaction.

**Table 4:** Paired changes in immunologic, renal, hepatic, and lipid parameters from switch visit to 48 weeks. Values are reported as median (IQR). Paired changes are reported as median change (IQR). *p*-Values were calculated using the Wilcoxon signed-rank test.

	Switch Visit Median (IQR)	48 Weeks Median (IQR)	Median Paired Change (IQR)	*p*-Value
CD4+ T-cell count	740 (503–969)	766 (510.3–920)	5 (−119 to 104)	0.837
CD4+ T-cell percentage	36.8 (23.9–42.6)	35.7 (28.1–41.9)	0 (−1.7 to 2.8)	0.712
CD8+ T-cell count	769.5 (551.8–1139.8)	748.5 (581.8–1094.2)	3.5 (−164.4 to 173.6)	0.914
CD8+ T-cell percentage	35.8 (27–45.2)	35.1 (28–46)	−1 (−3 to 2)	0.376
CD4/CD8 ratio	1.1 (0.6–1.6)	1.1 (0.7–1.5)	0.01 (−0.27 to 0.1)	0.251
Creatinine	0.9 (0.8–1)	0.9 (0.8–1.1)	0 (−0.09 to 0.03)	0.221
eGFR	94 (74–98)	93.2 (77.8–100)	0 (−2 to 7.5)	0.099
AST	26 (21–34.5)	29.5 (21.2–41.2)	2 (−2 to 9.2)	0.085
ALT	25 (22–38)	31 (22–55)	6 (−4 to 14)	**0.008**
GGT	24 (17.5–40.5)	22 (18–36.5)	1 (−14.5 to 8)	0.656
Total cholesterol (paired *n* = 49)	199 (162–230)	165 (142–198)	−22 (−52 to 1)	**<0.001**
HDL-C	48 (38–61)	44 (39–52)	−3 (−9 to 2)	**0.022**
LDL-C (paired *n* = 46)	118 (88–153)	106.5 (80.8–133.2)	−13.5 (−29.8 to 5.8)	**0.001**
Triglycerides (paired *n* = 50)	112.5 (70.8–169.5)	94 (73.5–131.2)	−10.5 (−54 to 13.8)	**0.021**
Non-HDL cholesterol	149 (121–180)	125 (99–157)	−25 (−49.5 to −4.5)	**<0.001**
Total Cholesterol/HDL-C ratio	4.2 (3.5–5.4)	3.8 (3.3–4.4)	−0.45 (−0.83 to 0.31)	**0.008**
LDL-C/HDL-C ratio	2.4 (2.1–3)	2.2 (1.8–2.7)	−0.14 (−0.47 to 0.39)	0.190
Triglycerides/HDL-C ratio	2.2 (1.3–4.6)	1.9 (1.5–3.7)	−0.34 (−1.41 to 0.29)	**0.038**

Table 1 reports baseline characteristics among all participants with available data at the switch visit. Table 4 reports paired analyses and therefore includes only participants with available measurements both at switch and at 48 weeks for each variable; consequently, switch-visit medians may differ slightly from those reported in Table 1. Bold values indicate statistically significant *p* values (*p* < 0.05). Abbreviations: ALT, alanine aminotransferase; AST, aspartate aminotransferase; CD4, cluster of differentiation 4; CD8, cluster of differentiation 8; eGFR, estimated glomerular filtration rate; GGT, gamma-glutamyl transferase; HDL-C, high-density lipoprotein cholesterol; IQR, interquartile range; LDL-C, low-density lipoprotein cholesterol. T-cell counts are reported as cells/µL; creatinine as mg/dL; eGFR as mL/min/1.73 m^2^; AST, ALT, and GGT as IU/L; lipid concentrations as mg/dL; lipid ratios are unitless.

## Data Availability

The data supporting the findings of this study are not publicly available because they contain information that could compromise participant privacy. Data may be made available from the corresponding author upon reasonable request and with appropriate approvals.

## Supplementary Materials

The following supporting information can be downloaded at https://www.mdpi.com/article/10.3390/idr18050105/s1; Table S1: Availability of follow-up data and paired observations; Table S2: Paired changes from switch to 24 weeks; Table S3: Paired changes from 24 to 48 weeks; Table S4: Median paired lipid changes from switch visit to 48 weeks according to previous NRTI backbone; Figure S1: Median paired lipid changes from switch visit to 48 weeks according to previous NRTI backbone.

## Abbreviations

The following abbreviations are used in this manuscript:

3TC	Lamivudine
ABC	Abacavir
ADR	Adverse drug reaction
ALT	Alanine aminotransferase
ART	Antiretroviral therapy
AST	Aspartate aminotransferase
ATV	Atazanavir
BIC	Bictegravir
CD4	Cluster of differentiation 4
CD8	Cluster of differentiation 8
CKD-EPI	Chronic Kidney Disease Epidemiology Collaboration
DDI	Drug-drug interaction
DOR	Doravirine
DOR/3TC/TDF	Doravirine/lamivudine/tenofovir disoproxil fumarate
DRV/c	Darunavir/cobicistat
DTG	Dolutegravir
EFV	Efavirenz
eGFR	Estimated glomerular filtration rate
EVG/c	Elvitegravir/cobicistat
FTC	Emtricitabine
GGT	Gamma-glutamyl transferase
HBsAg	Hepatitis B surface antigen
HBV	Hepatitis B virus
HCV	Hepatitis C virus
HDL-C	High-density lipoprotein cholesterol
HIV	Human immunodeficiency virus
INSTI	Integrase strand transfer inhibitor
IQR	Interquartile range
LDL-C	Low-density lipoprotein cholesterol
NNRTI	Non-nucleoside reverse transcriptase inhibitor
NRTI	Nucleoside/nucleotide reverse transcriptase inhibitor
NVP	Nevirapine
PI	Protease inhibitor
PWH	People with HIV
RAL	Raltegravir
RPV	Rilpivirine
TAF	Tenofovir alafenamide
TDF	Tenofovir disoproxil fumarate
