# Supplementary material for: Virologic, Lipid, Renal and Hepatic Laboratory Outcomes After Switching to Doravirine/Lamivudine/Tenofovir Disoproxil Fumarate in Treatment-Experienced People with HIV: Real-World Evidence from the SHINe-SHIC Cohort
Source: Infect Dis Rep. 2026 Sep 17;18(5):105. doi: 10.3390/idr18050105 (PMC13600043; doi:10.3390/idr18050105)
Supplement: Supplementary file 1 [file idr-18-00105-s001.zip › idr-4411989-supplementary.pdf]

Table S1. Availability of follow-up data and paired observations

Variable	Switch available, *n*	24 weeks available, *n*	48 weeks available, *n*	Paired switch-24w, *n*	Paired switch-48w, *n*	Paired 24-48w, *n*
**HIV RNA**	95	74	81	73	79	66
**CD4+ T-cell count**	90	63	79	58	73	55
**CD8+ T-cell count**	90	61	76	56	70	51
**CD4/CD8 ratio**	87	58	77	51	70	49
**Serum creatinine**	74	63	62	49	55	47
**eGFR**	72	56	62	41	53	40
**AST**	61	52	44	39	38	33
**ALT**	71	53	54	44	49	41
**GGT**	39	29	37	21	27	22
**Total cholesterol**	67	55	62	41	49	43
**HDL-C**	60	52	60	41	47	45
**LDL-C**	59	53	58	40	46	44
**Triglycerides**	69	62	58	47	50	45
**Non-HDL cholesterol**	56	45	58	34	43	39
**Total cholesterol/HDL-C ratio**	56	45	58	34	43	39
**LDL-C/HDL-C ratio**	59	52	58	40	46	44
**Triglycerides/HDL-C ratio**	59	52	58	41	46	44

Note: Paired observations indicate the number of participants with available measurements at both compared timepoints. Non-HDL cholesterol and lipid ratios were considered available only when all required component lipid measurements were available.

Table S2. Paired changes from switch to 24 weeks

parameter	Switch visit Median (IQR)	24 weeks Median (IQR)	Median (IQR) paired change	p value
**HIV RNA**	0 (0-19)	0 (0-19)	0 (0 to 0)	0.876
**CD4+ T-cell count**	682 (460.3-928.5)	675.7 (491.2-990)	13.6 (-40.2 to 124.5)	0.138
**CD4+ T-cell percentage**	36.4 (24.5-41.2)	35.9 (26.4-40.3)	-0.2 (-2 to 2.7)	0.923
**CD8+ T-cell count**	769.5 (545.3-1110.4)	768 (514.8-1015.5)	26.5 (-140 to 136.6)	0.654
**CD8+ T-cell percentage**	37 (29.9-46.9)	39 (28-44.9)	0 (-2.1 to 2.2)	0.899
**CD4/CD8 ratio**	1.1 (0.6-1.4)	1.1 (0.6-1.3)	-0.02 (-0.22 to 0.06)	0.106
**Creatinine**	0.9 (0.8-1)	0.8 (0.8-0.9)	-0.01 (-0.1 to 0.01)	0.160
**eGFR**	95 (78.1-99.2)	98 (86-107)	3 (-1 to 12)	**0.030**
**AST**	25 (20-32)	31 (25.5-38)	5 (-0.5 to 12)	**0.001**
**ALT**	25 (21-38)	36.5 (22.8-50.2)	4 (-1 to 17.2)	**0.011**
**GGT**	25 (18-42)	28 (17-49)	1 (-3 to 8)	0.600
**Total cholesterol**	209 (162-244)	179 (152-210)	-19 (-49 to 2)	**< 0.001**
**HDL-C**	47 (40-61)	47 (38-60)	0 (-5 to 6)	0.728
**LDL-C**	121.5 (94-158.5)	122 (87.5-136.2)	-2.5 (-25.2 to 14)	0.342
**Triglycerides**	122 (77.5-170)	103 (70.5-147.5)	-10 (-53 to 7.5)	**0.020**
**Non-HDL cholesterol**	154.5 (126.2-188.8)	130.5 (108.5-162.5)	-18.5 (-49.5 to 2.5)	**0.001**
**TC/HDL ratio**	4.3 (3.7-5.6)	3.8 (3.3-4.7)	-0.45 (-0.97 to 0.1)	**0.006**
**LDL/HDL ratio**	2.4 (2.1-3.3)	2.1 (1.6-2.8)	-0.21 (-0.61 to 0.01)	**0.008**
**TG/HDL ratio**	2.3 (1.4-4.3)	2.1 (1-3.3)	-0.42 (-1.43 to 0.07)	**0.008**

Table S3. Paired changes from 24 to 48 weeks

	24 weeks, median (IQR)	48 weeks, median (IQR)	Median paired change (IQR)	p value
**HIV RNA**	0 (0 to 19)	0 (0 to 28.8)	0 (0 to 0)	0.855
**CD4+ T-cell count**	674 (466.4 to 1007)	753 (458 to 923)	0 (-66 to 77.9)	0.884
**CD4+ T-cell percentage**	35.5 (25.8 to 40.5)	33.8 (28.2 to 40.8)	0.57 (-2 to 3)	0.420
**CD8+ T-cell count**	672 (466.5 to 992.5)	679 (592 to 1026.7)	0 (-124.1 to 80.7)	0.790
**CD8+ T-cell percentage**	36.3 (27.6 to 43.7)	34.8 (27.5 to 43.3)	-1 (-3.4 to 0.8)	0.056
**CD4/CD8 ratio**	1 (0.5 to 1.4)	1 (0.6 to 1.4)	0.02 (-0.09 to 0.1)	0.607
**Creatinine**	0.8 (0.8 to 0.9)	0.9 (0.8 to 1)	0 (-0.06 to 0.09)	0.371
**eGFR**	97 (83.8 to 110.8)	94.8 (78.6 to 104.2)	-0.75 (-6.78 to 3)	0.223
**AST**	30 (24 to 38)	28 (21 to 38)	-2 (-7 to 2)	0.146
**ALT**	34 (22 to 51)	31 (22 to 57)	3 (-6 to 9)	0.418
**GGT**	27 (20 to 38.8)	24.5 (19 to 30.8)	-1 (-5.75 to 5.75)	0.733
**Total cholesterol**	179 (157 to 207)	176 (142 to 206.5)	0 (-11.5 to 9.5)	0.936
**HDL-C**	47 (38 to 58)	44 (41 to 56)	0 (-4 to 4)	0.624
**LDL-C**	110.5 (86 to 133.5)	102 (84.5 to 127.2)	0 (-21.25 to 8.9)	0.139
**Triglycerides**	88 (70 to 139)	91 (67 to 119)	0 (-26 to 16)	0.910
**Non-HDL cholesterol**	126 (104.5 to 149.5)	123 (99 to 155.5)	0 (-16.5 to 7.5)	0.571
**TC/HDL ratio**	3.7 (3.2 to 4.3)	3.5 (3.1 to 4)	-0.04 (-0.36 to 0.23)	0.233
**LDL/HDL ratio**	2 (1.6 to 2.5)	2.1 (1.7 to 2.4)	-0.01 (-0.17 to 0.24)	0.851
**TG/HDL ratio**	2 (1.2 to 3.2)	1.8 (1.3 to 2.8)	0.02 (-0.6 to 0.51)	0.920

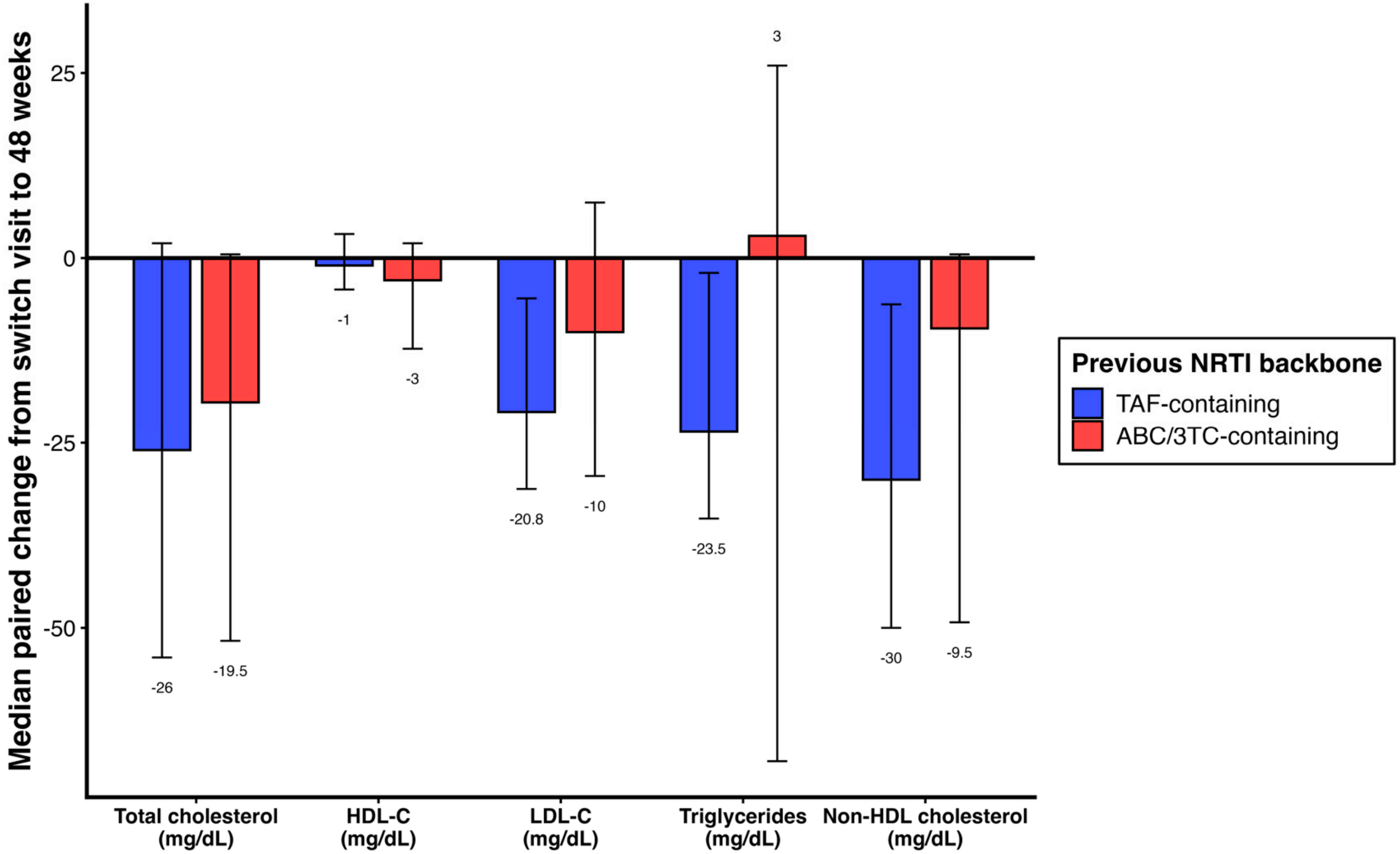

Figure S1. Median paired lipid changes from switch visit to 48 weeks according to previous NRTI backbone. Bars represent median paired changes and error bars represent interquartile ranges. Values above or below bars indicate median change. The figure displays the two main informative switch groups, TAF-containing and ABC/3TC-containing regimens, both switching to TDF-containing DOR/3TC/TDF. Participants previously receiving TDF-containing regimens are not shown because no TDF exposure change occurred and the subgroup was too small for meaningful graphical representation. Abbreviations: ABC, abacavir; HDL-C, high-density lipoprotein cholesterol; IQR, interquartile range; LDL-C, low-density lipoprotein cholesterol; NRTI, nucleoside/nucleotide reverse transcriptase inhibitor; TAF, tenofovir alafenamide; TDF, tenofovir disoproxil fumarate.

Table S4. Median paired lipid changes from switch visit to 48 weeks according to previous NRTI backbone

Parameter	Previous NRTI backbone	n	Median paired change (IQR)
Total cholesterol (mg/dL)	TAF-containing	20	-26 (-54 to 2)
	ABC/3TC-containing	26	-19.5 (-51.75 to 0.5)
	TDF-containing	1	-22 (-22 to -22)
	Unknown/not classifiable	2	-38 (-44 to -32)
LDL-C (mg/dL)	TAF-containing	16	-20.8 (-31.25 to -5.45)
	ABC/3TC-containing	27	-10 (-29.5 to 7.5)
	TDF-containing	1	-4.6 (-4.6 to -4.6)
	Unknown/not classifiable	2	-25.5 (-33.25 to -17.75)
HDL-C (mg/dL)	TAF-containing	16	-1 (-4.25 to 3.25)
	ABC/3TC-containing	28	-3 (-12.25 to 2)
	TDF-containing	1	0 (0 to 0)
	Unknown/not classifiable	2	-1.5 (-1.75 to -1.25)
Triglycerides (mg/dL)	TAF-containing	18	-23.5 (-35.25 to -2)
	ABC/3TC-containing	29	3 (-68 to 26)
	TDF-containing	1	-85 (-85 to -85)
	Unknown/not classifiable	2	-55 (-64.5 to -45.5)
Non-HDL cholesterol (mg/dL)	TAF-containing	16	-30 (-50 to -6.25)
	ABC/3TC-containing	24	-9.5 (-49.25 to 0.5)
	TDF-containing	1	-22 (-22 to -22)
	Unknown/not classifiable	2	-36.5 (-42.25 to -30.75)
Total cholesterol/HDL-C ratio	TAF-containing	16	-0.51 (-0.92 to -0.23)
	ABC/3TC-containing	24	-0.12 (-0.87 to 0.45)
	TDF-containing	1	-0.54 (-0.54 to -0.54)
	Unknown/not classifiable	2	-0.73 (-0.77 to -0.7)
LDL-C/HDL-C ratio	TAF-containing	16	-0.33 (-0.57 to -0.07)
	ABC/3TC-containing	27	-0.02 (-0.4 to 0.41)
	TDF-containing	1	-0.11 (-0.11 to -0.11)
	Unknown/not classifiable	2	-0.46 (-0.56 to -0.35)
Triglycerides/HDL-C ratio	TAF-containing	15	-0.47 (-1.05 to -0.26)
	ABC/3TC-containing	28	0.1 (-0.83 to 0.81)
	TDF-containing	1	-2.07 (-2.07 to -2.07)
	Unknown/not classifiable	2	-1.39 (-1.76 to -1.02)

Note: Values are median paired changes from switch visit to 48 weeks, with interquartile ranges. Previous NRTI backbone was categorized according to the regimen used immediately before switch. Results are descriptive only and no formal between-group comparison was performed. The TDF-containing group was very small and represented TDF-to-TDF switches, with no change in TDF exposure. Abbreviations: ABC, abacavir; HDL-C, high-density lipoprotein cholesterol; IQR, interquartile range; LDL-C, low-density lipoprotein cholesterol; NRTI, nucleoside/nucleotide reverse transcriptase inhibitor; TAF, tenofovir alafenamide; TDF, tenofovir disoproxil fumarate.
